# The genetic basis for survivorship in coronary artery disease

**DOI:** 10.3389/fgene.2013.00191

**Published:** 2013-09-27

**Authors:** Jennifer R. Dungan, Elizabeth R. Hauser, Xuejun Qin, William E. Kraus

**Affiliations:** ^1^School of Nursing, Duke University, DurhamNC, USA; ^2^Section of Medical Genetics, Department of Medicine, Duke University, DurhamNC, USA; ^3^Durham Epidemiologic Research and Information Center, Durham Veterans Affairs Medical Center, DurhamNC, USA; ^4^Department of Medicine, Cardiology, Duke University, DurhamNC, USA

**Keywords:** survivorship, survival, novel phenotype, coronary artery disease, atherosclerosis, *LSAMP* gene, cardioprotection

## Abstract

Survivorship is a trait characterized by endurance and virility in the face of hardship. It is largely considered a psychosocial attribute developed during fatal conditions, rather than a biological trait for robustness in the context of complex, age-dependent diseases like coronary artery disease (CAD). The purpose of this paper is to present the novel phenotype, *survivorship in CAD* as an observed survival advantage concurrent with clinically significant CAD. We present a model for characterizing survivorship in CAD and its relationships with overlapping time- and clinically-related phenotypes. We offer an optimal measurement interval for investigating survivorship in CAD. We hypothesize genetic contributions to this construct and review the literature for evidence of genetic contribution to overlapping phenotypes in support of our hypothesis. We also present preliminary evidence of genetic effects on survival in people with clinically significant CAD from a primary case-control study of symptomatic coronary disease. Identifying gene variants that confer improved survival in the context of clinically appreciable CAD may improve our understanding of cardioprotective mechanisms acting at the gene level and potentially impact patients clinically in the future. Further, characterizing other survival-variant genetic effects may improve signal-to-noise ratio in detecting gene associations for CAD.

“Old age is not a disease – it is strength and survivorship, triumph over all kinds of vicissitudes and disappointments, trials and illnesses.”

– Maggie Kuhn

## Introduction

Survivorship is a unique clinical construct that can be characterized by the intersection of temporal factors related to lifespan, disease-related burden and treatment, and mortality. The term survivorship connotes traits or conditions of maintaining survival, whereas survival characterizes the state of living. Coronary disease continues to be a leading cause of death in the U. S. and a significant source of rising disease burden (Roger et al., 2012), despite a decline in cardiovascular disease-related mortality in past decades as a result of improved knowledge of risk factors and biomarkers, and advances in pharmacotherapeutics and coronary interventions (McGovern et al., 2001). Moreover, clinical *prediction* of survival likelihood in the setting of coronary artery disease (CAD) is inaccurate and difficult. Given recent advances in the identification of cardioprotective gene variants, biological markers may provide critical insight into the conditions that support survival in the context of CAD, or, what we term, *survivorship in CAD*. The purpose of this paper is to introduce and define a novel phenotype, “survivorship in CAD,” to provide a review of the literature, to present hypotheses for genetic contributions, and to present preliminary evidence of survival-variant genes unique to CAD.

### Survival vs. survivorship

The concept of survivorship has many related terms and definitions, depending upon the context and the field. “Survival” is most commonly used as an epidemiological construct denoting a period of time between an event and a “failure.” The medical community considers survival to be the length of time from medical intervention (e.g., coronary artery bypass surgery, stent placement, initiation of aspirin) to an event such as death (i.e., failure). The primary medical goal is to evaluate effectiveness in the prevention of mortality, and this model implies that the disease state is known or diagnosed. Mullan (1985) states that people become survivors at the time they are diagnosed with a life-threatening disease. In a *New England Journal of Medicine* essay, he explained, “Survival … begins at the point of diagnosis because that is the time when cancer patients are forced to confront their own mortality and begin to make adjustments that will be part of their immediate, and to some extent, long-term future” (Mullan, 1985, 271). Survivor- “ship” implies that a trait or attribute is possessed related to survival, such as in the case of cancer survivors exhibiting strength and perseverance throughout their treatment and in the confrontation of death (Zebrack, 2000). While the term “survivorship” most often has a psychosocial connotation of sustaining life during hardship, we hypothesize that biologic robustness can be a trait favoring survival even in the context of complex diseases such as coronary disease.

**Figure 1:**
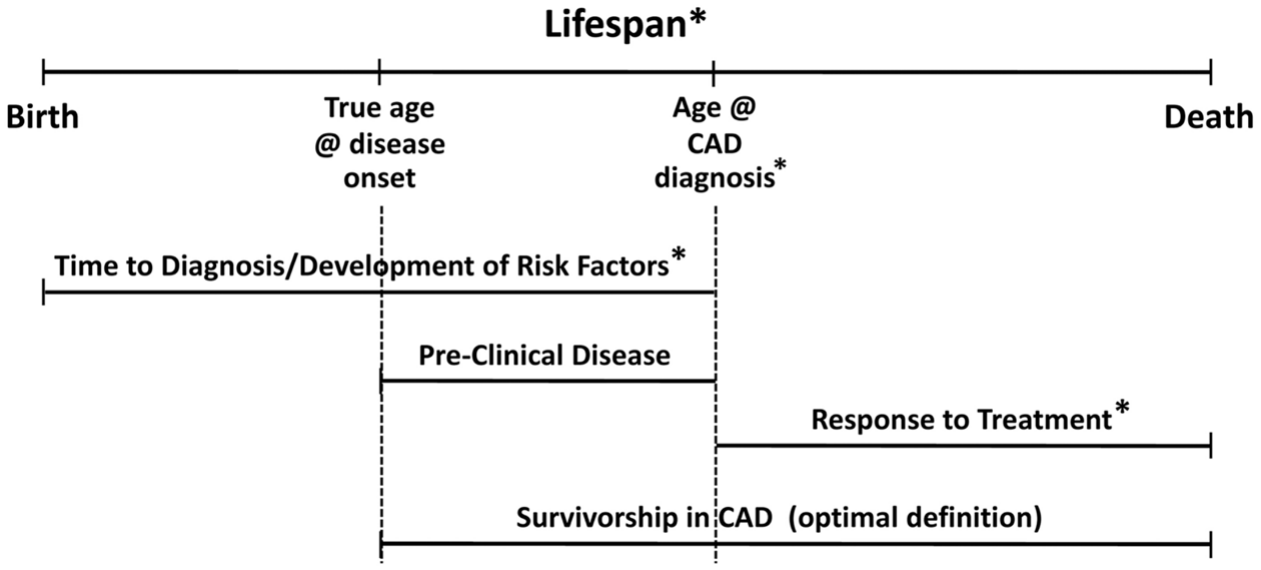
**Model for survivorship in CAD depicting temporal relationships with clinical constructs.** Asterisks indicate established genetic effects in the literature.

The gerontology literature refers to survivorship as lifespan longevity irrespective of health indices (Murabito et al., 2012); and, many researchers in aging consider longevity to be *healthy* survival to old age. Centenarians are exemplars of longevity, but some data suggest that nearly one-third of centenarians have had age-related morbidities for 15 or more years (Terry et al., 2008), making at least some of those survivors apparently robust to the effects of pathophysiologic insults—perhaps due to some biological advantages. Some researchers have begun to explore the hypothesis that centenarians are genetically predisposed to physiological states that are protective against heart disease (Grimaldi et al., 2006) and other conditions, perhaps via “buffered disease genes” that promote longevity by “buffering” genetically determined age-related diseases (Bergman et al., 2007). However, *these people are different from those with known coronary disease who survive despite their disease*, who may potentially embody distinct genetic characteristics. These caveats lead us to make an important distinction about defining the construct of survivorship in CAD. Timing and context are everything. We present a model (Figure 1) that accounts for temporal, clinical, and genetic interrelationships that characterize survivorship in CAD. In our goal to determine an optimal definition for survivorship in CAD and determine the plausibility of genetic contribution to this phenotype, we considered the interplay of these factors. We present a theory-based definition for survivorship in CAD as a survival advantage concurrent with clinically significant CAD and propose an optimal measurement of this phenotype as the time at initial biological atherosclerotic disease onset to time of coronary-related death. We discuss the caveats of observing and measuring the phenotype as recommended and offer alternatives for approximating the construct of survivorship in CAD through the use of overlapping phenotypes. We hypothesize that genes may contribute to the survivorship in CAD phenotype.

### The hypothesis of genetic contribution to survivorship in CAD

The natural history of CAD makes elucidating genetic contributions to “survivorship” a complex challenge. Our model (Figure 1) helps to illustrate the interplay of these factors in generating our hypothesis. Survivorship in CAD is only one of a number of related and overlapping CAD phenotypes. Genetic contributions to these overlapping phenotypes suggest that there may be shared genetic markers for survivorship in CAD. Moreover, there could be different but correlated genetic effects at each stage, reflected by genetic heterogeneity and/or differential magnitudes of genetic effect across the time-disease continuum. Recent findings identify genetic associations with risk variants in cardiovascular-related mortality and sudden cardiac death (see extensive review by Arking and Sotoodehnia, 2012). However, the survivorship in CAD phenotype hinges on the distinction between determining risk for CAD-related mortality events vs. characterizing the propensity to survive beyond clinical expectation with significant CAD (as in, cardioprotection). The key is being able to parse out the proportion of overlapping genetic variance that contributes specifically to survivorship in the context of CAD. To lend additional support for hypothesized genetic involvement in the survivorship in CAD phenotype, we look to genetic variants associated with known cardioprotection phenotypes—both *in-vitro* and limited *in-vivo* evidence, reviewed later in the paper.

Genetic heterogeneity likely drives marked inter-individual variation in disease initiation, progression, and response to treatment, thus, is likely to affect survival likelihood. Naghavi and SHAPE task force members (Naghavi et al., 2006) provide insight into the variability therein: “At every level of risk factor exposure, the amount of established atherosclerosis and the vulnerability of actual events varies greatly, probably because of genetic variability in an individual's susceptibility to atherosclerosis and propensity to arterial thrombosis (“vulnerable blood”) and ventricular arrhythmias (“vulnerable myocardium”)”, (Naghavi et al., 2006, 4H). Coupled with great variance in modifiable risk factor profiles, the task of finding biological variants and/or biomarkers that pinpoint the initiation of CAD is a daunting one. The following are necessary for accurately defining the origin of CAD in the study of this novel phenotype: (1) a consensus for what constitutes “the” biological/biochemical *initiation* of CAD; (2) a sensitive and reliable biomarker for that initial biological disease state; and, (3) evidence that the particular biomarker(s) independently predict development of clinically appreciable CAD. In estimating the earliest known point of origin for disease initiation (i.e., implications 1 and 2), the epidemiological tenets of what is “detectable” in the preclinical phase of disease are currently being challenged by scientific advances, which could make future research for their prediction of the development of CAD (and survivorship in CAD) more viable. We next present a review of the evidence for genetic involvement in each of the presented phases in our model and reflect on the impact of temporal contexts of the CAD continuum to the study of the genetics of survivorship in CAD.

### The temporal stages of biology and disease: identifying the survivorship in CAD point of origin

Our lifespans are a complex continuum of biological and environmental interactions that result in various stages of wellness and decline (and/or disease). In Figure 1, the lifespan stages are depicted by the top horizontal line progressing from birth to death. Disease progression is also by nature a temporal process with multiple clinical stages, represented here by the middle horizontal lines. Specific to CAD, the major clinical stages can be considered: development of risk factors/preclinical disease, symptomatic stage with/without diagnosis and/or coronary event(s); treatment/maintenance, and death. Inter-individual variance in disease presentation and the heterogeneous nature of CAD affect the onset and length of these stages, and, inherently the way that CAD phenotypes are defined and studied. Given aforementioned definitions, “survivorship in CAD” could simply be defined as survival time from point of diagnosis to death. While this definition suits investigations of medical treatment, genetic investigations seeking to identify markers associated with survival as a function of biologic protection from CAD-related mortality should consider an alternate definition, specifically, beginning with a better and earlier point of origin.

The time point at which we define the origin of the survival curve is critical. In survival analyses, the origin refers to the natural point in time in which the person becomes “at risk.” If CAD diagnosis were used as the origin for the survivorship in CAD phenotype, this would be an inaccurate assessment because the pre-clinical phase of CAD also corresponds to a period where risk for death from CAD is known. Specifically, it is estimated that the *initial presentation* of CAD is sudden cardiac death (SCD) for at least 20–30% of cases (Myerburg and Junttila, 2012; Roger et al., 2012). SCD cases demonstrate risk of death from asymptomatic CAD during the pre-clinical/pre-diagnosis phase and there is evidence of genetic variants associated with the risk for CAD-associated sudden death (Westaway et al., 2011). Therefore, the optimal origin for defining the survivorship in CAD phenotype should be the *initial* biological disease state, which we know occurs before the diagnosis of CAD. Methodologically, this is currently problematic. We cannot “carbon-date” the exact onset of disease, given the potentially long period of pre-clinical, asymptomatic phase associated with CAD. Current proxies are traditional biomarkers, such as coronary artery calcium (CAC) scores, C-reactive protein (CRP), and carotid intima media thickness (CIMT), discussed later.

### Development of risk factors/pre-clinical disease

Development of risk factors and pre-clinical disease overlaps with our defined phase of survivorship in CAD and likely shares genetic contributions to this phenotype. Here, we consider both traditional (age, diabetes, smoking, hypertension, dyslipidemia, obesity, and family history of early-onset CAD) and non-traditional risk factors. The multitude of non-traditional risk factors include, for example, CRP, homocysteine, fibrinogen, lipoprotein a, calcium score, metabolic syndrome, renal disease, and microproteinuria. Evidence of genetic contribution to these well-studied risk factors has been established and summarized extensively in the literature to date.

For any disease, the pre-clinical phase is characterized by three periods: the initiation of biological insult, the un-detectable pre-clinical state, and the detectable pre-clinical state (Herman et al., 2002). Pre-clinical coronary disease is characterized by the development of risk factors and the physiologic initiation of inflammation and atheroma development. An ideal marker for approximating the initial biological disease state for survivorship in CAD would be one that had high sensitivity and specificity for detecting initial endothelial dysfunction (oxidation of low-density lipoprotein in the arterial wall and/or inflammation of endothelial cells). Genetic contribution to these broad physiologic phases warrants a separate review. Briefly, some strong sub-clinical CAD biomarker candidates exist for the earliest known phases of atherosclerotic development (Table 1). Accurate detection of the earliest initiation of the biophysiologic process of disease development appears to be on the horizon. The “traditional” subclinical markers of vascular disease (CRP, CAC, and CIMT) have evidence of moderate heritability. Heritability estimates for CRP levels is estimated between 26 and 45%, depending on the population studied (MacGregor et al., 1999; Pankow et al., 2001; Lange et al., 2006; Fox et al., 2008). The heritability of quantity of CAC within carotid vessels of asymptomatic white individuals has been estimated between 38 and 42% (Cassidy-Bushrow et al., 2007; Rampersaud et al., 2008) after adjustment for various risk factors. Cassidy-Bushrow et al. (2007) further demonstrated heritability of progression of CAC across 7 years, with a post-adjustment estimate of 40%, wherein genetic factors explained 14% of the variation in CAC progression. The heritability of CIMT was first demonstrated in the Framingham Offspring cohort, with age- and sex-adjusted estimates of 37–44% (Fox et al., 2003). Estimates of twin cohorts (Zhao et al., 2008; Lee et al., 2012) are higher with adjusted *h*^2^ of 38–59%, and as much as 65% (adjusted *h*^2^) in Caribbean Hispanic populations (Sacco et al., 2009).

**Table 1:** **Sub-clinical CAD biomarkers**.

**Atherosclerotic phase of involvement**	**Biomarkers**	**Review citation**
Oxidation of low-density lipoprotein in the arterial wall	OxLDL, OxPL/ApoB	Fraley and Tsimikas, 2006
Inflammation of endothelial cells	CRP, IL-6, WBC, VCAM-1, ICAM-1, PLA2	Galkina and Ley, 2007; Vaccarino et al., 2011

OxLDL, oxidized low-density lipoprotein; OxPL, oxidized phospholipid; ApoB, apolipoprotein B; CRP, C-reactive protein; IL-6, interleukin-6; WBC, white blood cells; VCAM-1, vascular cellular adhesion molecule-1; ICAM-1, intracellular adhesion molecule-1; PLA2, phospholipase A2.

Pre-clinical disease can begin as early as childhood, especially where traditional risk factors are present in early ages. The presence of raised fibrous plaques has been documented in children as young as 8 years of age who had type 1 diabetes and who died from accidental causes in the Bogalusa Heart Study (Berenson et al., 1998). Other epidemiologic studies of children and young adults (Pathobiological Determinants of Youth Study, Cardiovascular Risk in Young Finns; Coronary Artery Risk Development in Young Adults) have documented the presence of coronary and aortic fatty streaks in these populations and correlations between detectable streaks and some traditional risk factors (McMahan et al., 2006; Loria et al., 2007; Hartiala et al., 2012). The fact that the biological origin of CAD may begin so early in life constitutes another layer of complexity in defining the point of origin for survivorship in CAD; yet, advances may be leading us to more accurate detection of the initial biophysiologic insults leading to CAD, as early as childhood. Given the potential for emerging biomarkers to characterize the earliest detectable initiation of atherosclerotic disease (earlier than CRP, CAC, and CIMT), we assert that the construct of survivorship in CAD remains best defined as survival from time at initial biological atherosclerotic disease onset to time of coronary-related death. We believe that biomarker advances will allow us to optimally define earlier points of origin in the near future. If such biomarkers (CRC, CAC, CIMT, or novel) are measured in an at-risk, pre-clinical population, the survivorship in CAD origin could be the time at biomarker capture (for those with biomarker levels corresponding to increased risk for CAD). Participants could be followed from pre-clinical phase to point at conversion to CAD diagnosis, then until coronary-related death. Capture of genetic data from this type of cohort would be ideal for investigating the genetic contribution to survivorship in CAD. Furthermore, one could test the genetic contributions to each phase or point of survival along the time-disease-treatment trajectory. This approach could discriminate whether identified genes are equally important at each phase or whether there are different genes corresponding to different effects across this continuum. Until earlier biomarkers are validated, or until an at-risk cohort (with sufficient data and power) is prospectively followed as previously described, proxies for the origin point, such as time of clinical CAD diagnosis, will be necessary.

### Symptomatic stage with/without CAD diagnosis

CAD diagnosis is driven by symptomatology. For those whose initial symptoms are less severe or who survive their initial atherosclerotic vascular event, numerous candidate genes have been associated with the presence and diagnosis of CAD. Recently, Yoo et al. (2012) explored the role of polymorphisms in the *Rho-associated kinase 2* (*ROCK-2*) gene in vasospastic angina and found that a 5-marker haplotype conferred protection from coronary vasospasm in 106 Korean adults undergoing coronary angiography (*p* = 0.007). Genetic investigation into CAD symptoms is new territory in the literature but the demonstration of genetic association with vasospasm identifies the potential for genetic variants to be involved in the predisposition to CAD symptoms. Age of onset of CAD diagnosis has strong evidence of genetic contributions to development of CAD, with earlier age of onset suggesting a stronger genetic effect (Arking et al., 2003; Hauser et al., 2004; Connelly et al., 2006; Shah et al., 2006, 2009). Estimates of over 2000 known or suspected candidate genes involved in CAD are present in the literature (IBC 50K CAD Consortium, 2011). The 9p21 region has the most evidence established to date for CAD, as the most highly replicated variants come from the 9p21 region for significant associations with CAD and myocardial infarction (Palomaki et al., 2010), and sudden and arrhythmic cardiac death (Newton-Cheh et al., 2009).

### Treatment/maintenance

The treatment and maintenance phase of the CAD continuum primarily includes interventional cardiology procedures and/or pharmacological management. Genetic contribution to treatment response in CAD is also well-established, particularly as the pharmacogenomics revolution has been the fastest pipeline for translation of genetic screening in the context of complex, multifactorial diseases. A recent review by Voora and Ginsburg (2012) summarizes the evidence of genetic associations with the most common cardiovascular drug classes used in the prevention and treatment of CAD (antiplatelet agents, warfarin, statins, beta-blockers, diuretics, and antiarrhythmic drugs). Significant pharmacogenetic associations have been reported for mortality. This is key to our construct for two reasons: first, cardiovascular treatment effects and the survivorship in CAD phenotype could share genetic variation—further supporting our hypothesis; and, second, there is a likelihood that in situations where non-shared genetic effects are present for survivorship in CAD, cardiovascular treatment effects (involving genetic risk or not) may independently bias survival models. Similar caveats persist related to treatment effects from interventional procedures for CAD, such as with coronary artery bypass grafting (CABG), percutaneous coronary angioplasty (PTCA), and stent placement, for which patients may also have varied prognoses and outcomes based on certain genetic markers (Muehlschlegel et al., 2010; Cayla et al., 2011; Lobato et al., 2011).

### Death

A landmark analysis of clinician vs. computational prognoses of survival by Kong and colleagues (Kong et al., 1989) indicated that clinically, we are poor at predicting survival in CAD; and, that accurate estimation of survival outcomes is difficult. Over 20 years later, strong evidence has been found implicating the following as independent predictors of death in CAD: age, race (Thomas et al., 2010), presence of comorbidities (diabetes/metabolic syndrome), renal disease, hypertension (Emerging Risk Factors Collaboration et al., 2010), depression (Whang et al., 2010), and reduced ejection fraction (Movahed and Sattur, 2010; Kuhl et al., 2011). In those at-risk or diagnosed with CAD, the use of aspirin, beta-blockers, ACE-inhibitors, and statins can significantly reduce the risk of death by 15–50%; early reperfusion can reduce mortality by 25–30%.

Genetic factors may provide further insight into mortality risk. Evidence for genetic involvement in mortality phenotypes such as in sudden cardiac death (Arking and Sotoodehnia, 2012), acute coronary syndrome (Xu et al., 2013), and all-cause mortality post-acute coronary syndrome (Morgan et al., 2008, 2011) has been modestly demonstrated. Aouizerat et al. (2011) conducted a GWAS of sudden cardiac death in patients with CAD and reported 11 significant gene associations for six novel candidates and validated eight known variants for this phenotype. Johnson and colleagues (Johnson et al., 2012) have identified multiple fatty acid gene variants associated with survival to admission and survival to discharge in out-of-hospital sudden cardiac death. The relationship between SCD and survivorship is testable. If there is a risk allele for SCD, we could expect the frequency of that allele to be lower in survivors of CAD.

### Caveat: cardioprotective genes

Given our distinction regarding survivorship in CAD as the propensity to survive beyond clinical expectation with significant CAD, it is logical that the focus of finding unique genetic variants for survivorship could be on cardioprotective genetic variants rather than cardiovascular risk markers. It is also important to consider the potential for heterozygous advantage for survival conferred by some risk variants for these overlapping constructs, as in the example of malaria and sickle cell disease. Some promising data exist on cardioprotective genes, but the majority of the science in this area is limited to animal models and *in-vitro* studies of cellular survival of cardiac cells and/or prevention of apoptosis (Zhang et al., 2009; Weng et al., 2010). Innate cardioprotective phenotypes include ischemic preconditioning, hypoxic preconditioning, and heat shock preconditioning. Work in this area has uncovered some promising candidate genes, most prominently the *nuclear factor kappa-B* (*NFKB*) (Wilhide et al., 2011) and related *heat shock protein* (*HSP*) genes, necessary mediators of cardioprotective mechanisms following ischemic preconditioning (Tranter et al., 2010). Micro-RNAs are hypothesized to be involved in cardioprotection due to their ability to regulate processes involved in cardiac injury and protection (see review by Kukreja et al., 2011). While other biomarkers have been implicated in preconditioning phenotypes [hypoxia inducible factor-1 (HIF-1; Tekin et al., 2010; Ong and Hausenloy, 2012); tissue kallikrein (TK; Messadi-Laribi et al., 2008); erythropoietin, heme oxygenase-1, and inducible nitric oxide synthase (Ong and Hausenloy, 2012), paroxonase, clusterin, and apolipoprotein A-1 [(MacKness et al., 1997)], there is limited evidence for genetic involvement in cardioprotection.

Population-based studies have identified some candidate genes significantly associated with protection against incident CAD, but have failed to produce candidate genes for survival in CAD. Briefly, A single nucleotide polymorphism (rs3217989) corresponding to cyclin-dependent kinase inhibitor-2B (*CDKN2B*) in the 9p21 region was protective against incident CAD in a sample of 548 African Americans [*OR* = 0.19, 95% *CI* = 0.07–0.50, *p* = 0.0008, a finding that was further replicated in a larger combined sample of 990 African Americans (Kral et al., 2011)]. The ADA^*^2 allele in the adenosine deaminase (*ADA*) gene was hypothesized by Safranow et al. (2007) to modulate cardioprotection via its indirect effects on levels of adenosine—a potent cardioprotective agent. They reported lower frequencies of the ADA^*^2 gene variant in CAD-diagnosed individuals in a sample of 371 Poles (Safranow et al., 2007).

### Alternative hypotheses

We hypothesize that genes could play a unique role in the ability to survive in the context of clinically significant coronary disease. Genetic principals that offer support for survival traits in the context of unfavored phenotypes are heterozygous advantage and antagonistic pleiotropy. For example, evaluation of the Framingham Heart Study revealed that *APOE* gene variants were associated with survival-related pleiotropic effects in cancer and age of onset of CAD (Kulminski et al., 2011). An alternative hypothesis is that there is no genetic involvement in coronary disease-related survival. As we have presented earlier, up to one-third of centenarians have age-related morbidities (such as heart disease) for 15 or more years (Terry et al., 2008). Furthermore, we have presented evidence of genetic contribution to overlapping phenotypes with survivorship in CAD and hypothesize shared genetic variance among these phenotypes. Other non-genetic factors could contribute more significantly to survival (such as treatment effects) that may mask or override any genetic effects contributing to survivorship in CAD. Observations of greater survival in the context of CAD could be also be due to a population effect in which a select group (or family) has a tendency for longer survival in spite of the presence of CAD (theoretically, as in family-based centenarians from Mediterranean areas).

## Pilot evidence of survival-variant genes in CAD using a proxy definition for survivorship in CAD

As we are unaware of a cohort for which pre-clinical biomarkers and genetic data have been captured for at-risk, asymptomatic people prospectively followed through development of CAD that has adequately powered mortality rates, we set out to generate at least preliminary evidence of genetic variation in survival likelihood for symptomatic CAD-diagnosed people. We performed a secondary analysis of 1885 subjects from a primary case-control genetics study of symptomatic patients undergoing cardiac catheterization (Catheterization Genetics Study; CATHGEN), described elsewhere (Sutton et al., 2008). Briefly, the primary study recruited patients presenting to the cardiac catheterization lab (regardless of disease status) for a cardiovascular genetics study and biorepository in which medical history, clinical data, and biological samples were collected. Biological data were stored in the Center for Human Genetics facility; all other data were stored in the Duke Databank for Cardiovascular Disease and maintained at the Duke Clinical Research Institute (Fortin et al., 1995). Both the primary and secondary studies obtained approval from the Duke University Medical Center Institutional Review Board. All participants provided informed consent for participation. Our specific aim for the secondary analysis was to evaluate whether the likelihood of survival was significantly different among candidate genes for cases with angiographically-defined, clinically significant CAD. For this case-only analysis, we evaluated 34 previously genotyped single nucleotide polymorphisms (SNPs; Table 2), representing five *a priori* CAD candidate genes of interest from the primary CATHGEN study. The candidate genes for our secondary analyses were either previously associated with age-related CAD phenotypes or were highly suspect of having survival effects, based on published findings from our group [*ALOX5AP* (Shah et al., 2008; Crosslin et al., 2009), *FAM5C* (Connelly et al., 2006), *KALRN* (Wang et al., 2007), *LSAMP* (Wang et al., 2008), *PLA2G7* (Sutton et al., 2008)]. Genomic DNA for CATHGEN was extracted from whole blood using the Puregene system (Gentra Systems, Minneapolis, MN, USA). Genotyping was performed at the Duke Center for Human Genetics genotyping laboratory (Durham, NC, USA). The primary study originally selected SNPs in 2004 based on reported literature for CAD and age-related CAD candidate genes, and from a list of age-related CAD candidate SNPs in the GENECARD family study of early-onset CAD (Hauser et al., 2004). Additional follow-up SNPs in these candidate genes were later selected based on the aims of ancillary studies and/or availability of the SNPs for these candidate genes on selected genotyping platforms. Three different genotyping platforms were used in the primary study: TaqMan 7900 HT (Applied Biosystems, New York, USA); Illumina HumanOmni1-Quad_v1-0_C chip; and, custom Illumina GoldenGate Bead Arrays (San Diego, CA, USA). The 384-well plates included a total of 20 quality control samples (eight CEPH (Center d'Étude du Polymorphisme Humain) pedigree individuals, eight study sample duplicates, and four no-template controls). SNP mismatches were reviewed by an independent genotyping supervisor for potential genotyping errors. Each SNP had a call frequency across all individuals of at least 95%; each individual had a call rate across all SNPs of at least 95%. For the TaqMan platform, blinded duplicate samples were used to determine the error rates, which were <0.2% among SNPs that passed genotyping quality control. For the secondary analysis, we selected SNPs for these candidate genes that met our quality control metrics and that were also genotyped at that time in the Framingham Heart Study (as we were anticipating replication analyses in that dataset), leaving the 34 SNPs in Table 2 for this secondary pilot analysis. All SNPs met our established criteria (Shah et al., 2009; Zhang et al., 2010) for quality control (QC) Hardy-Weinberg equilibrium (HWE) and linkage disequilibrium (LD).

**Table 2:** **SNPs**.

**Gene**	**SNPs**
*ALOX5AP*	rs3803277, rs4147064, rs9506352
*FAM5C*	rs12076854, rs2990996, rs1442569, rs16832305, rs1272400
*KALRN*	rs7616435, rs627830, rs9289235, rs7623685, rs2289843, rs1867647, rs2289420
*LSAMP*	rs9822311, rs10511352, rs6776244, rs1870709, rs9847048, rs1518898, rs6788787, rs1915585, rs1462845, rs4356827, rs1354152, rs1698041, rs2937673, rs1676232, rs1381801, rs4404477, rs4687889
*PLA2G7*	rs1421378, rs1862008

ALOX5AP, arachidonate 5-lipoxygenase-activating protein; FAM5C, family with sequence similarity 5, member C; KALRN, kalirin; LSAMP, limbic system-associated membrane protein; PLA2G7, phospholipase A2; group VII (platelet-activating factor acetylhydrolase, plasma).

Statistical analyses were performed using the R program's Survival package (R Development Core Team, 2008). Means and frequencies were calculated for demographic variables, diagnosis, and events. The survivorship in CAD phenotype was defined in this analysis as time from angiographically-defined CAD case status in symptomatic individuals to time at all-cause mortality. The primary study did not recruit asymptomatic patients with CRP, CAC, or CIMT data to serve as an optimal point of origin for evaluation of survivorship in CAD. We considered the alternative analytical approach in which we would identify a subset of control subjects that converted to CAD diagnosis (to use as a proxy for the pre-symptomatic point of origin); however, only two participants would have met the criteria for the analyses. More importantly, by our recommendation, CRP, CAC, and CIMT levels would be ideal for verifying the increased risk for CAD in these “converters,” but these labs are not routinely ordered on patients that meet “control” status after cardiac catheterization. An excess of missing data for the cause of death variable precluded our use of the ideal phenotype endpoint of coronary-related death. Therefore, we used time from cardiac catheterization to all-cause mortality as a proxy definition. In order to determine if there were significant genetic effects on survival in the context of CAD but not in controls, we performed analyses stratified by CAD case status. Thus, Cox proportional hazards models estimated instantaneous risk [hazard ratio and 95% confidence interval (CI)] of all-cause mortality by genotype groups separately in CAD cases and controls censored on number of days from study enrollment (time at coronary catheterization) to all-cause death or last follow-up. Survival curves for long-term survival were illustrated using Kaplan-Meier curves. Cases were defined as having at least one major epicardial vessel having at least 75% stenosis on coronary angiography (Duke CAD index > 23) (Sutton et al., 2008). Controls were defined as having no appreciable CAD (Duke CAD index < 23), corresponding to angiographic data indicating no more than one major epicardial vessel having less than or equal to 75% occlusion as demonstrated by coronary angiography, and no documented history of cerebrovascular or peripheral vascular disease, myocardial infarction, transplant, or interventional or surgical coronary revascularization procedures (Sutton et al., 2008). An additive inheritance model was assumed, assigning wild-type genotypes a value of 0, heterozygous genotypes a value of 1, and risk homozygous genotypes a value of 2. All SNPs were analyzed separately with a basic model evaluating only the main effect of genotype and a separate covariate model controlling for age, sex, body mass index (BMI), and histories of smoking, type 2 diabetes, hyperlipidemia and hypertension. As a *post-hoc* analysis for the case-only group, we added CAD index as a covariate in the full clinical covariate model in order to determine if the significant survival effects were impacted by disease severity. In addition, we evaluated the impact of sex on the survival results using a stratified Cox proportional hazards analyses in CAD cases, controlling for age, body mass index (BMI), and histories of smoking, type 2 diabetes, hyperlipidemia and hypertension.

For this pilot analysis, 1885 subjects with genetic data meeting standard quality control metrics were analyzed. Demographic and cohort characteristics are presented in Table 3. Four hundred subjects (21.2%) were deceased on follow-up at the time of analysis; 1155 subjects met our criteria for CAD diagnosis with an event rate of 21.9% (*n* = 253) and 730 were considered controls, with an event rate of 20.1% (*n* = 147). Vital events were confirmed through the National Death Index, as previously described (Shah et al., 2010). As expected, CAD cases who experienced events tended to be older, had a greater burden of disease (higher CAD index), lower BMI, and a higher frequency of hypertension. Deceased CAD cases also had a lower frequency of hyperlipidemia, demonstrating a paradoxical phenomenon between mortality and hyperlipidemia, previously reported by our group and likely due to treatment effects or confounders (Wang et al., 2009; Shah et al., 2012). Deceased participants with CAD also had a reduced frequency of smoking compared to living CAD cases, which may be explained by the self-report measurement of history of smoking and/or the option to report having ever smoked or currently smoking as positive history vs. not currently smoking as negative history. Racial representation varied little by event and diagnosis groups.

**Table 3:** **CATHGEN demographic and clinical characteristics**.

**Characteristic**	**All (*N* = 1885)**	**All subjects (*N* = 1885)**		**CAD cases (*N* = 1155)**	
		**Alive (*n* = 1485)**	**Dead (*n* = 400)**	**Alive (*n* = 902)**	**Dead (*n* = 253)**
Age, $\bar{x}\pm SD$, (range)	62.8 ± 13.0 (20–93)	61.3 ± 12.6 (24–91)	68.4 ± 12.9 (20–93)	59.1 ± 12.8 (27–90)	67.6 ± 13.1 (34–91)
CAD index, $\bar{x}\pm SD$	41.4 ± 31.3	40.0 ± 30.9	47.0 ± 32.3	59.9 ± 21.5	66.7 ± 21.2
Male, no (%)	1210 (64.2)	946 (63.7)	264 (66)	667 (74)	183 (72.3)
Race, no (%)					
White	1409 (75.0)	1104 (74.3)	305 (76.3)	683 (75.7)	198 (78.3)
Black/AA	338 (18.0)	270 (18.2)	68 (17)	159 (17.6)	38 (15.0)
Other	138 (7.0)	111 (7.5)	27 (6.7)	59 (6.7)	17 (6.7)
Body mass index, $\bar{x}\pm SD$	29.5 ± 6.6	29.9 ± 6.6	28.4 ± 6.4	30.0 ± 6.6	28.7 ± 5.8
Smoking, no (%)	1013 (53.8)	796 (53.6)	217 (54.3)	555 (61.5)	143 (56.5)
Dyslipidemia, no (%)	1173 (62.2)	931 (62.7)	242 (60.5)	650 (72.1)	177 (70.0)
Hypertension, no (%)	1322 (70.1)	1022 (68.8)	300 (75)	634 (70.3)	204 (81)

CAD, coronary artery disease; SD, standard deviation; AA, African American.

Based on a follow-up of 2738 days (about 7 years, 5 months), three SNPs (rs1462845, rs1915585, and rs6788787) in the *LSAMP* gene had significant hazards of death in CAD cases (Table 4, uncorrected *p*-values), but differed in their gene dosing patterns and directions of effect. Data for these three SNPs met the assumption of proportional hazards (data not shown). Minor allele frequencies are presented in Table 4 and genotype frequencies by sample and case status are presented in Table 5. For the rs1462845 SNP, the risk of death for CAD cases was 1.24 times greater for each addition of the minor (risk) allele compared to wild-type genotype (*p* = 0.044). For rs1915585 and rs6788787, the hazard ratios are less than one (with significant *p*-values; *p* = 0.044 and 0.037, respectively), suggesting that the minor alleles may have a significant protective effect against risk of death in CAD. The genotype effects on survival remained significant when controlling for known CAD risk factors (Table 4). In order to determine whether the survival effects for genotype were specifically driven by CAD, we then performed previously described survival analyses in control subjects. None of the control subjects had significant gene effects on survival in either model, suggesting the genotype effects on survival may be unique to CAD.

**Table 4:** **Cox proportional hazards by SNP for CAD cases and controls**.

**SNP**	**MA (MAF)**	**CAD cases (G) HR (95% CI)**	**Controls (G) HR (95% CI)**	**CAD cases (C) HR (95% CI)**	**Controls (C) HR (95% CI)**
rs1462845	G (0.15)	1.24 (1.00–1.54)^*^	1.26 (0.89–1.79)	1.26 (1.02–1.57)^*^	1.10 (0.76–1.59)
rs1915585	T (0.39)	0.70 (0.49–0.99)^*^	0.99 (0.57–1.70)	0.64 (0.45–0.91)^*^	1.04 (0.59–1.84)
rs6788787	A (0.15)	0.70^*^ (0.50–0.98)^*^	0.99 (0.57–1.70)	0.68 (0.49–0.94)^*^	1.22 (0.70–2.15)

SNP, single nucleotide polymorphism; MA, minor allele; MAF, minor allele frequency; (G), main genotype model; (C), covariate model; HR, hazard ratio; CI, confidence interval;

* p < 0.05.

**Table 5:** **Genotype frequencies by sample and case status**.

**Sample**	**rs1462845**			**rs1915585**			**rs6788787**		
	**AA**	**AA**	**GG**	**GG**	**GT**	**TT**	**GG**	**GA**	**AA**
Full (*N* = 1885)	0.38	0.46	**0.16**	0.71	0.27	**0.02**	0.72	0.26	**0.02**
CAD Cases (*n* = 1155)	0.39	0.46	**0.15**	0.73	0.25	**0.02**	0.72	0.25	**0.03**
Controls (*n* = 730)	0.37	0.44	**0.19**	0.71	0.28	**0.01**	0.71	0.28	**0.01**

Risk homozygous genotype frequencies indicated in bold.

**Figure 2:**
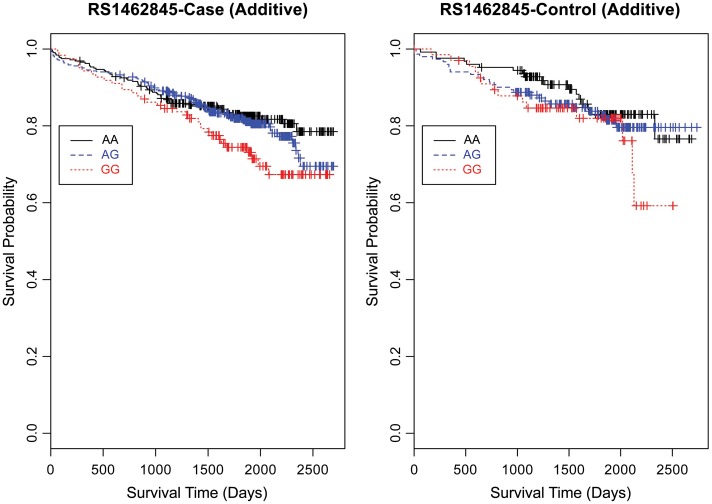
**Kaplan–Meier survival curves for CAD cases vs. controls in additive (genotype) model for *LSAMP* SNP rs1462845.** *X*-axis displays the number of days from index catheterization to death (all-cause mortality). *Y*-axis displays the Kaplan-Meier survival probability by genotype. G is the minor allele; AA, wild-type genotype (reference; black curve); AG, heterozygous genotype (blue curve); and GG, risk homozygous genotype (red curve).

**Figure 3:**
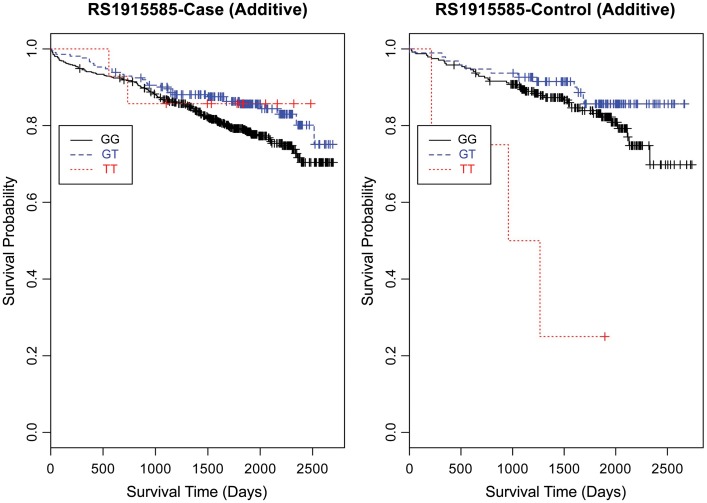
**Kaplan-Meier survival curves for CAD cases vs. controls in additive (genotype) model for *LSAMP* SNP rs1915585.** *X*-axis displays the number of days from index catheterization to death (all-cause mortality). *Y*-axis displays the Kaplan-Meier survival probability by genotype. T is the minor allele; GG, wild-type genotype (reference, black curve); GT, heterozygous genotype (blue curve); and TT, risk homozygous genotype (red curve).

**Figure 4:**
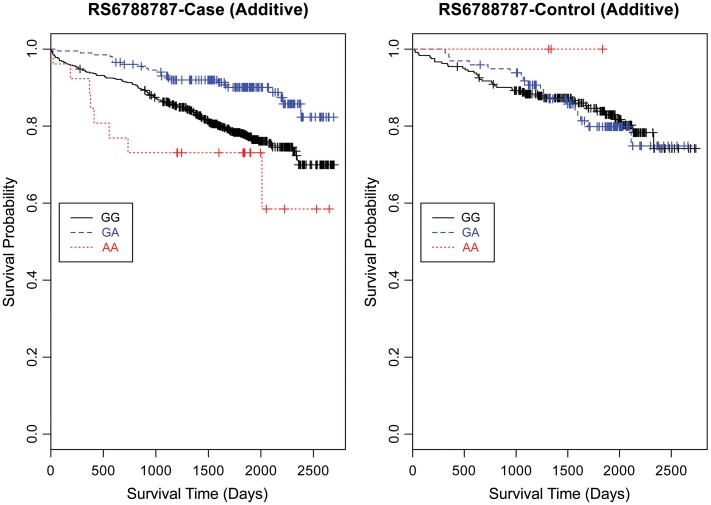
**Kaplan–Meier survival curves for CAD cases vs. controls in additive (genotype) model for *LSAMP* SNP rs6788787.** *X*-axis displays the number of days from catheterization to death (all-cause mortality). *Y*-axis displays the Kaplan–Meier survival probability by genotype. A is the minor allele; GG, wild-type genotype (reference; black curve); GA, heterozygous genotype; and AA, risk homozygous genotype (red curve).

Evaluating the Kaplan-Meier (K-M) survival curves for CAD cases revealed an expected gene dosing pattern for rs1462845 (Figure 2). In an additive genetic model of survival, we would expect to see a gene-dosing phenomenon, whereby having two copies of the risk allele (risk homozygous genotype) confers the worst survival, having no copies of the risk allele (wild-type homozygous genotype) confers the best survival, and having one copy of each (heterozygous genotype) falls between the two. For rs1915585 (Figure 3) and rs6788787 (Figure 4), K-M curves revealed that subjects with the heterozygous genotype (blue line) conferred better survival than those with the wild-type genotype, which explains the hazard ratios of less than 1 for these SNPs. These results could be due to the low frequency of risk homozygous carriers for the rs1915585 and rs6788787 variants in our sample, the result of insufficient power or rare homozygosity (see Table 5). Another possible explanation for observing this phenomenon is heterozygous advantage. Heterozygous advantage (or, hybrid vigor) can be the result of dominance or overdominance in the population, which acts to selectively advantage the heterozygous individual in spite of the presence of a single copy of the risk allele. These SNPs do not have documented evidence of hybrid vigor at this time. Other possible explanations for this inappropriate gene-dosing could be antagonistic pleiotropy or survival bias. Others have simulated erosion of genetic associations for highly lethal diseases (such as myocardial infarction) due to culling of risk variants from the population (Anderson et al., 2011). As aforementioned, longitudinal data would allow for evaluation of whether the surviorship in CAD effects are due to genetic factors or are observed as a result of survival bias. It is important to note that traditional survival bias would be considered a loss of genetic information due to mortality events in the data; yet, we are interested in characterizing a genetically-driven net survival advantage in the context of CAD. While our pilot data are not designed to show exact proof of the latter concept, our results do lend preliminary support for genetic differences in survival unique to symptomatic CAD, adding to the theoretical basis for genetic involvment in the survivorship in CAD phenotype.

**Figure 5:**
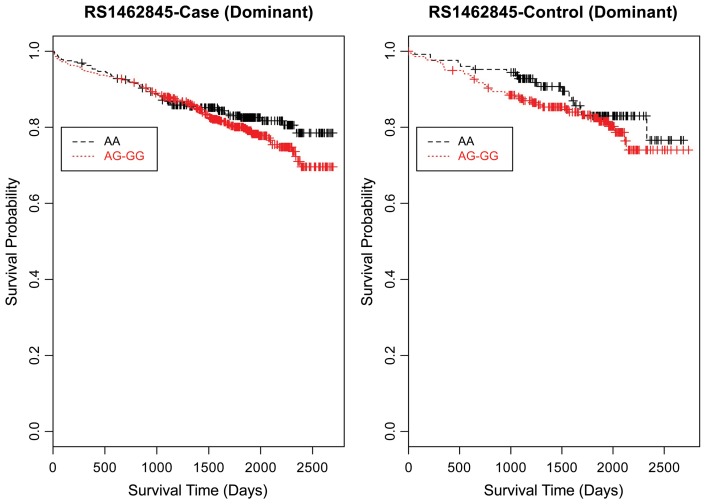
**Kaplan–Meier survival curves for CAD cases vs. controls in dominant (allele) model for *LSAMP* SNP rs1462845.** *X*-axis displays the number of days from index catheterization to death (all-cause mortality). *Y*-axis displays the Kaplan–Meier survival probability by genotype. G is the minor allele; AA, wild-type genotype (reference; black curve), and red curve indicates AG and GG genotypes combined.

**Figure 6:**
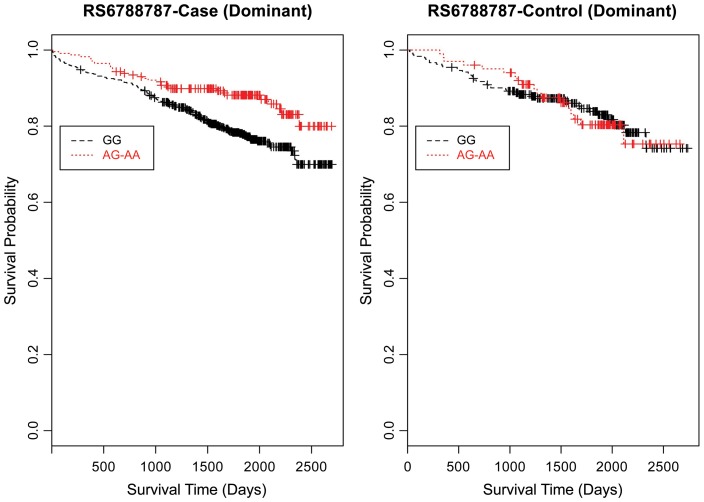
**Kaplan–Meier survival curves for CAD cases vs. controls in dominant (allele) model for *LSAMP* SNP rs6788787.** *X*-axis displays the number of days from catheterization to death (all-cause mortality). *Y*-axis displays the Kaplan-Meier survival probability by genotype. A is the minor allele; GG, wild-type genotype (reference; black curve); and red curve indicates GA and AA genotypes combined.

**Figure 7:**
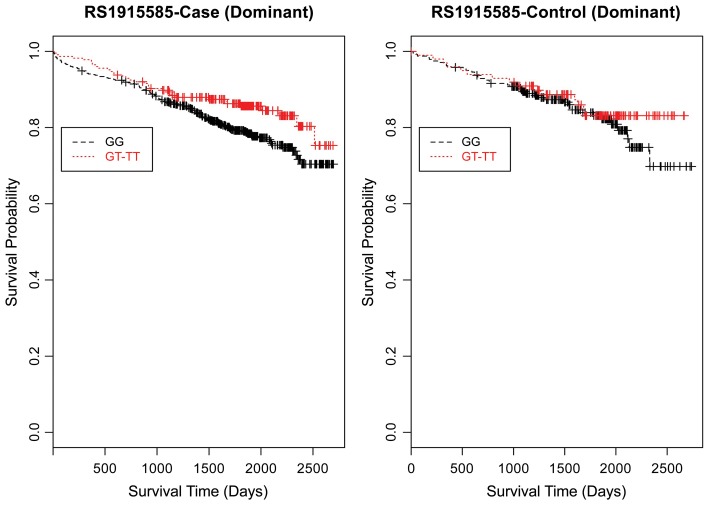
**Kaplan–Meier survival curves for CAD cases vs. controls in dominant (allele) model for *LSAMP* SNP rs1915585.** *X*-axis displays the number of days from index catheterization to death (all-cause mortality). *Y*-axis displays the Kaplan-Meier survival probability by genotype. T is the minor allele; GG, wild-type genotype (reference; black curve); and red curve indicates GT and TT genotypes combined.

Furthermore, when examining the dominant model, (a common approach to improve power to detect an association when the minor allele frequency is low), the K-M curve for rs1462845 showed expected curves, wherein having any copy of the minor (risk) allele confers worse survival compared to the wild-type genotype (Figure 5). However, combining risk genotypes in the dominant model for the rs6788787 SNP (Figure 6, red line) results in a survival curve that suggests the minor (risk) allele confers better survival in the presence of CAD. Theoretically, this could drive unexpected or spurious results in an association between the *LSAMP* SNP and CAD if survival effects were not considered. The SNP rs1915585 (Figure 7) showed a trend similar to rs6788787 for both additive and dominant models. To determine if the significant survival effects by genotype were due to the severity of CAD, we then performed the case-only Cox model, adding CAD index as a term in the full covariate model. The genotype effect remained significant for all three SNPs when controlling for disease severity (*p* = 0.03 for rs1462845, *p* = 0.01 for rs1915585, and *p* = 0.01 for rs6788787). CAD index itself was only marginally significant as a predictor of survival in this model (*p* = 0.07 for rs1462845 and rs1915585 and *p* = 0.04 for rs6788787). As sex differences are established in CAD and CAD-related mortality, we also evaluated Kaplan–Meier curves and Cox models for CAD cases stratified by sex, controlling for age, body mass index (BMI), and histories of smoking, type 2 diabetes, hyperlipidemia, and hypertension. All three SNPs had significant genotype effects in male CAD diagnosed subjects (*n* = 850) but not female CAD diagnosed subjects (*n* = 305; Supplemental Table 1). The rs1462845 survival curves (Supplemental Figure 1) demonstrated genotype-specific effects in males comparable to the full dataset and no appreciable genotype-specific pattern on survival in females. For the rs1915585 and rs6788787 SNPs (Supplemental Figures 2, 3), genotype-specific survival patterns for males and females were consistent with the full sample model (e.g., consistent heterozygous advantage effects), but conclusions about these effects cannot be drawn due to the reduced power in this sex-based analyses and reduced homozygosity similar to the full CAD case models (Supplemental Table 1). These results suggest sex-specific genotype effects on survival in symptomatic, CAD-diagnosed individuals for the rs1462845 SNPs and possible sex differences for other *LSAMP* SNPs; however, a larger sample size is necessary to confirm this relationship.

In summary, three *LSAMP* SNPs show significant differences in survival by genotype in CAD cases but not controls, even after adjusting for age, sex, body mass index (BMI), and histories of smoking, type 2 diabetes, hyperlipidemia, hypertension, and disease severity. Sex-stratified analyses revealed that these effects were unique to males. This work provides preliminary evidence of gene-related survival effects unique to CAD. The limbic system-associated membrane protein (*LSAMP*) gene is a 64–68 kD gene located on the long arm of chromosome 3. It encodes a neuronal surface glycoprotein [Online Mendelian Inheritance in Man (OMIM), 2011] and has been described as a tumor suppressor gene that may be fundamental to brain development (Wang et al., 2008). Our group has previously found multiple *LSAMP* SNPs to be significantly associated with late age of onset CAD (diagnosis in males ≥51 years of age, females ≥56 years of age), some of which had stronger genetic effects in individuals with severe CAD, i.e., the presence of left main coronary disease (Wang et al., 2008), further supporting *LSAMP*'s candidacy for CAD-specific survival effects. The rs6788787 and rs1915585 SNPs were previously reported to be in strong linkage disequilibrium in CATHGEN [pairwise r-square = 0.74 (Wang et al., 2008)], however, our subset sample data do not support LD between these two markers (all pairwise correlations less than 60%).

## Conclusion

Every minute, someone dies of coronary disease in America (Roger et al., 2011). The ratio of deaths per year to incident cases of stable angina is roughly equal (~400,000:500,000) (Roger et al., 2011). Investigating genetic contributions to improved survivorship with CAD could provide unique insights for better health promotion and prognosis. Identifying genetic variants associated with improved survivorship in CAD could lead to improved clinical prediction and insights into biological mechanisms critical to the complex disease-survival interface. Knowledge of such variants could also support a shift in focus from preventing death to promoting optimal conditions for survival in concert with known genetic makeup. Measurement of the optimal phenotype interval, consideration for shared and non-shared genetic effects with overlapping constructs, and interplay with treatment effects in this process will be critical components of such investigations. We hypothesize genetic contribution to this phenotype based on our model depicting shared genetic variation with related and overlapping constructs. We further support the concept of a genetic basis for survivorship in CAD with preliminary evidence of genetic differences in survival in CAD for certain *LSAMP* SNPs. This model can help generate hypotheses about the genetic architecture of survivorship in CAD and support appropriate measurement. Future work can address important questions about genetic and other factors involved in CAD-specific mortality and survival, which may help improve clinical prediction of survival and mortality in people with CAD. This could lead to further insight into the biological and/or functional mechanisms of survivorship in CAD. Finally, refining the genetic factors for survivorship in CAD may lead to improvement in the signal-to-noise ratio in genetic associations with CAD as well as for replications.

### Conflict of interest statement

The authors declare that the research was conducted in the absence of any commercial or financial relationships that could be construed as a potential conflict of interest.
