# Supplementary material for: The genetic basis for survivorship in coronary artery disease
Source: Front Genet. 2013 Sep 27;4:191. doi: 10.3389/fgene.2013.00191 (PMC3784965; doi:10.3389/fgene.2013.00191)
Supplement: Supplementary file 1 [file 59410__Data_Sheet_1.DOCX]

**Supplemental Table 1.** Genotype (additive model) effects on survival probability and frequencies, by gender. WT = wild-type homozygous genotype; Het = heterozygous genotype; RHom = risk homozygous genotype (bold).

| **SNP** | **Male CAD cases (*n* = 850)** | | | | **Female CAD cases (*n* = 305)** | | | |
| --- | --- | --- | --- | --- | --- | --- | --- | --- |
|  | **Gene (Additive)**  **p-value** | **Genotype Frequencies** | | | **Gene (Additive)**  **p-value** | **Genotype Frequencies** | | |
|  |  | WT | Het | **RHom** |  | WT | Het | **RHom** |
| rs1462845 | .04 | .40 | .46 | **.14** | .65 | .34 | .50 | **.16** |
| rs1915585 | .01 | .73 | .25 | **.02** | .64 | .73 | .26 | **.01** |
| rs6788787 | .01 | .71 | .25 | **.04** | .67 | .75 | .24 | **.01** |
