# Supplementary material for: The genetic basis for survivorship in coronary artery disease
Source: Front Genet. 2013 Sep 27;4:191. doi: 10.3389/fgene.2013.00191 (PMC3784965; doi:10.3389/fgene.2013.00191)
Supplement: Supplementary file 3 [file 59410__Presentation_2.PDF]

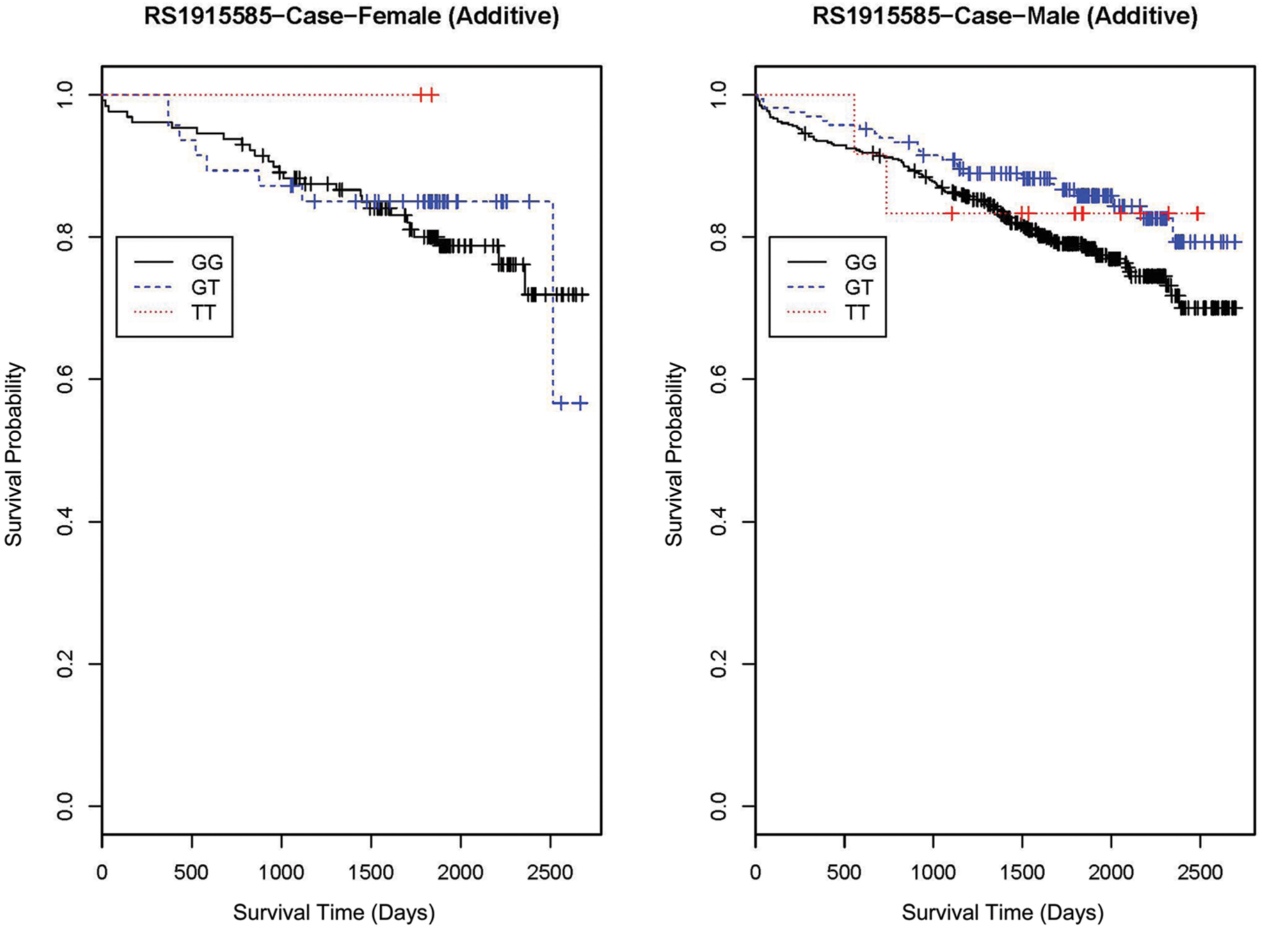

Supplemental Figure 2. Kaplan-Meier survival curves by gender for CAD cases in additive (genotype) model for *LSAMP* SNP rs1915585. X-axis displays the number of days from index catheterization to death (all-cause mortality). Y-axis displays the Kaplan-Meier survival probability by genotype. T is the minor allele; GG = wild-type genotype (reference; black curve), GT = heterozygous genotype (blue curve), and TT = risk homozygous genotype (red curve).
